# Comparison of efficacy of treatments for early syphilis: A systematic review and network meta-analysis of randomized controlled trials and observational studies

**DOI:** 10.1371/journal.pone.0180001

**Published:** 2017-06-28

**Authors:** Hong-ye Liu, Yan Han, Xiang-sheng Chen, Li Bai, Shu-ping Guo, Li Li, Peng Wu, Yue-ping Yin

**Affiliations:** 1 Reference STD Lab, National Center for STD Control, Chinese CDC, Institute of Dermatology, Chinese Academy of Medical Sciences, Peking Union Medical College, Jiangsu Key Laboratory of Molecular Biology for Skin Diseases and STIs, Nanjing, China; 2 Department of Dermatology and Venereology, First Affiliated Hospital of Shanxi Medical University, Taiyuan, China; 3 Health Statistics Teaching and Research Section, School of Public Health, Shanxi Medical University, Taiyuan, China; Azienda Ospedaliera Universitaria di Perugia, ITALY

## Abstract

**Background:**

Parenteral penicillin is the first-line regimen for treating syphilis, but unsuitable for some patients due to penicillin allergy and lacking health resources. Unfortunately, the efficacy of penicillin alternatives remains poorly understood. This study aimed to assess the efficacy of ceftriaxone and doxycycline/tetracycline in treating early syphilis relative to that of penicillin, and thereby to determine which antibiotic is a better replacement for penicillin.

**Method:**

By searching literature from PubMed, Cochrane Central Register of Controlled Trials, Embase, the Web of Science, and ClinicalTrials.gov and systematically screening relevant studies, eligible randomized controlled trials (RCTs) and observational studies on treatments with penicillin, doxycycline/tetracycline, and ceftriaxone for early syphilis were identified and combined in this systematic review. Estimated risk ratios (RRs) and 95% confidence intervals (CIs) were utilized to compare their serological response and treatment failure rates. At 12-month follow up, serological response rates were compared by a direct meta-analysis and network meta-analysis (NMA), while treatment failure rates were compared with a direct meta-analysis.

**Result:**

Three RCTs and seven cohort studies were included in this research. The results of NMA demonstrated that no significant differences existed in serological response rate at 12-month follow-up between any two of the three treatments (doxycycline/tetracycline vs. penicillin RR = 1.01, 95%CI 0.89–1.14; ceftriaxone vs. penicillin RR = 1.00, 95%CI 0.89–1.13; ceftriaxone vs. doxycycline/tetracycline RR = 0.99, 95%CI 0.96–1.03), which was consistent with the outcomes of the direct meta-analysis. In addition, the direct meta-analysis indicated that, at 12-month follow-up, penicillin and ceftriaxone treatment groups had similar treatment failure rates (RR = 0.92, 95%CI 0.12–6.93), while treatment failure rate was significantly lower among penicillin recipients than among doxycycline/tetracycline recipients (RR = 0.58, 95%CI 0.38–0.89).

**Conclusion:**

Ceftriaxone is as effective as penicillin in treating early syphilis with regard to serological response and treatment failure rate. Compared with doxycycline/tetracycline, ceftriaxone appears to be a better choice as the substitution of penicillin.

## Introduction

Syphilis, a multi-stage infectious disease, is caused by *Treponema pallidum subsp*. *pallidum* (*T*. *pallidum*) and usually transmitted sexually. Once a successful infection occurs, *T*. *pallidum* is capable of disseminating almost all tissues of the host where it may remain latent for a long period of time or induce protean clinical presentations. It can even penetrate human placenta, resulting in miscarriage, premature birth, stillbirth, or congenital syphilis. It is estimated by the World Health Organization (WHO) that there are 12 million new cases of syphilis globally every year, with 90% occurring in developing countries [1], but its incidence has also increased in North America and Western Europe where most of the cases involved men who have sex with men. Significantly, syphilis has been shown to contribute to the increased risk of acquisition and transmission of HIV infection [1–4]. Hence, this disease is a pivotal concern to public health globally.

Due to lacking an effective vaccine against syphilis, its treatment completely relies on antibiotics. Parenteral penicillin has been the first-line regimen for treating syphilis, which, however, is not accessible to patients in resource-limited settings where safe injection devices are not readily available. Furthermore, with its extensive use, the incidence of penicillin allergy is almost up to 10% [5]. Though recommended by some specialists [6–8], desensitization seems impractical in most of the primary care providers, since it has the risk of anaphylaxis, thereby requiring special emergency medical devices and drugs for rescue therapy. Therefore, patients with penicillin allergy have to appeal to alternative antibiotics. In this regard, azithromycin, ceftriaxone, and doxycycline/tetracycline have been employed as penicillin alternatives for many years [9]. However, in recent years, azithromycin-resistant strains of *T*. *pallidum*, which contain A2058G or A2059G mutation, have been reported in many countries and regions, resulting in clinical treatment failures there [10–12], which suggests that azithromycin is no longer suitable for treating syphilis worldwide, despite its high efficacy proved at one time [13, 14]. Currently, only ceftriaxone and doxycycline/tetracycline remain in the list of penicillin alternatives for syphilis therapy. However, the efficacy of penicillin alternatives in treating syphilis was assessed in very limited studies, some of which showed contradictory results [15]. Therefore, it remains unclear whether the alternative drugs differ in efficacy, although a few meta-analyses compared ceftriaxone and azithromycin with penicillin in terms of efficacy in treating early syphilis by using randomized controlled trials (RCTs) [16, 17], which demonstrated that their efficacy was not significantly different from that of penicillin. To date, no documented study has simultaneously assessed the efficacy of penicillin, ceftriaxone, and doxycycline/tetracycline in treating syphilis.

Network meta-analysis (NMA) is a key means to compare multiple interventions through integration of direct and indirect evidence [18]. This study aimed to assess the efficacy of ceftriaxone and doxycycline/tetracycline in the treatment of early syphilis relative to that of penicillin by using NMA, and thereby to determine which antibiotic is a better replacement for penicillin.

## Materials and methods

This research was performed according to the Preferred Reporting Items for Systematic Reviews and Meta-Analyses (PRISMA) guidelines (S1 Table).

### Search strategy

We carried out a systematic computerized literature search for RCTs and observational studies on patients who received penicillin, doxycycline, tetracycline, or ceftriaxone treatment for early syphilis. PubMed, Cochrane Central Register of Controlled Trials, Embase, the Web of Science, and ClinicalTrials.gov were searched from inception to June 30, 2016 by combining Medical Subject Headings (MeSH) descriptors with free text terms to identify relevant studies; and appropriate adjustment was made as database varied (S2 Table). The references of the included articles and documented meta-analyses were also retrieved manually to widen the scope of literature search.

### Study selection and data extraction

The eligible articles included in this study must meet the following criteria: (1) They are published RCTs or observational researches in English; (2) involved primary, secondary, or early latent syphilis; (3) made comparisons between penicillin and the alternatives (ceftriaxone, doxycycline/tetracycline), or between ceftriaxone and doxycycline/tetracycline in efficacy; and (4) provided adequate data on outcomes of interest.

The course of study selection, data extraction, and quality assessment of included studies was completed by two investigators independently. Any disagreements were settled by discussion. By scanning titles and abstracts of the searched studies, eligible ones were selected and their full texts were subsequently read. Excel database was used to extract the following information from included studies: first author, publication year, study type, stage of syphilis, intervention, and baseline characteristics. Outcomes of interest were serological response rates and treatment failure rates at both 6- and 12-month follow-up. Serological response was defined as the titer converting to negative or having a ≥ 4-fold (2 dilutions) decrease in Venereal Disease Research Laboratory Test/rapid plasma regain test/toluidine red unheated serum test (VDRL/RPR/TRUST) without increase during the follow-up period. Treatment failure was defined as clinical progression of the disease or having a ≥ 4-fold (2 dilutions) increase in VDRL/RPR/TRUST titer without an initial response during the follow-up period.

### Quality assessment of studies

The Cochrane risk of bias assessment tool was used to assess the quality of identified RCTs [19]. Review Manager 5.3 (Cochrane Collaboration, Oxford UK) was employed to generate the risk of bias figure. The Newcastle-Ottawa scale was adopted to evaluate observational studies [20].

### Statistical methods

Statistical analyses were performed using STATA 13.0 (College Station, Texas 77845, USA). Relative risk ratios (RRs), 95% confidence intervals (CIs), and prediction intervals (PrIs) were calculated for dichotomous variables. All probability values were two-tailed. *P* < 0.05 was considered statistically significant. Random effects model was used throughout the study [21]. Heterogeneity was examined by Q test [22] and *I*^*2*^ test [23]. *P*< 0.05 in Q test and *I*^*2*^ > 50% indicated statistically significant heterogeneity. If the results indicated heterogeneity existing, meta-regression was performed to identify its source [24]. A sensitivity analysis was utilized to determine if the pooled effects were robust. Inconsistency between direct and indirect evidence was assessed using the node-splitting approach proposed by Dias *et al*. [25], which separated the evidence concerning certain comparison into direct and indirect evidence. Egger’s [26] and Begg-Mazumdar tests [27] were used to evaluate publication bias indicators in a funnel plot.

## Results

### Study selection

**Fig 1:**
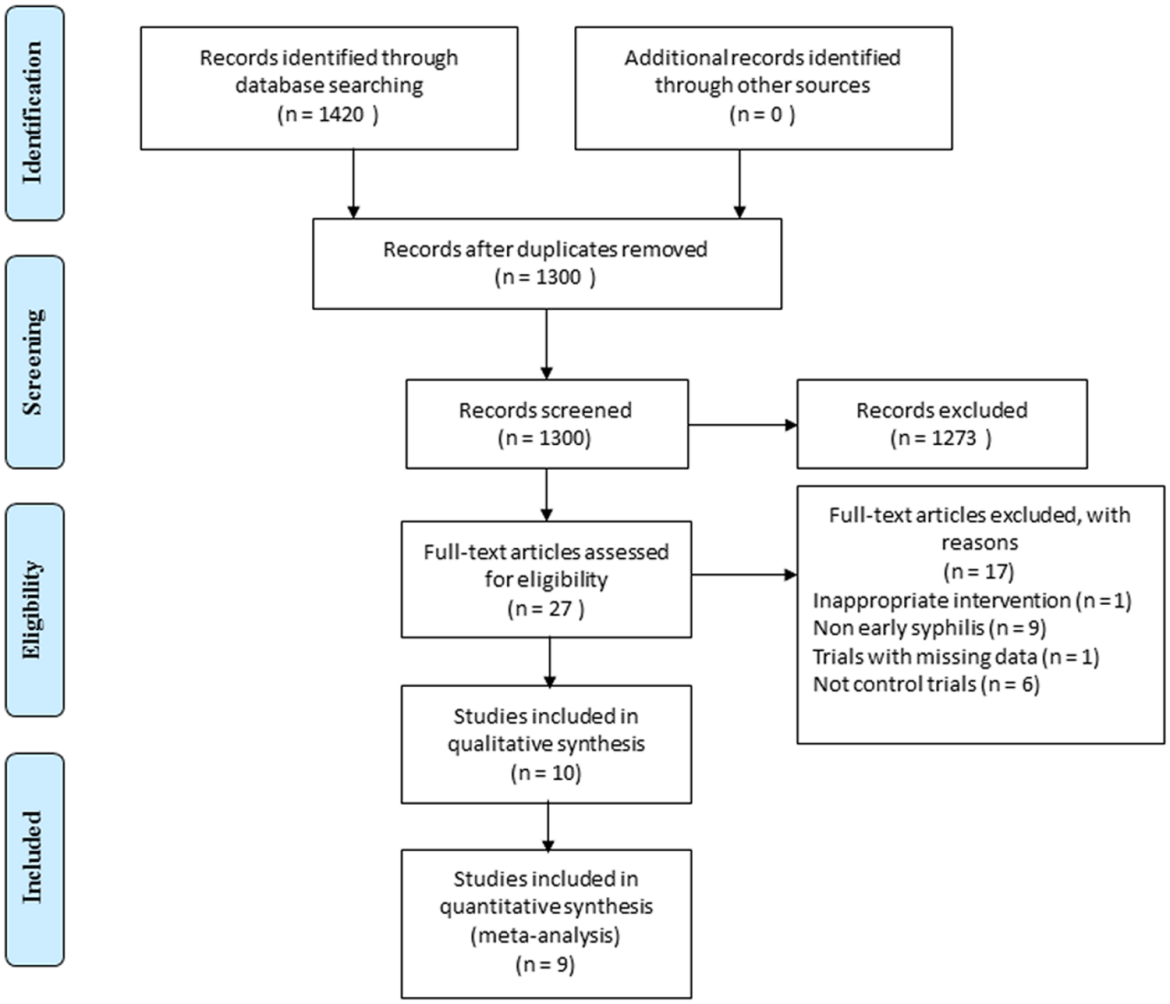
PRISMA flow diagram for article screening and selection process.

A total of 1420 citations were found. After removing duplicates, 1300 citations were screened by scanning their titles and abstracts; consequently, 1273 were excluded since they did not meet our inclusion criteria. By examining full texts of the remainder, 10 studies with outcomes of interest at 6- and 12-month follow-up were identified and included in this research (Fig 1). They encompassed three RCTs involving ceftriaxone vs. penicillin [28–30], and seven observational studies [31–37] including a three arms study that compared ceftriaxone and doxycycline with penicillin [33]. Regarding the data of follow-up, only five included studies referred to the records of serological tests at 6-month and nine studies at 12-month. Considering the inadequacy of the data of serological tests at 6-month follow-up, they were analyzed with a qualitatively descriptive study.

### Characteristics of included studies

A total of 2049 patients received treatments for early syphilis in the included studies, ranging in age from 15 to 80 years; 1281 were males (two studies [28, 36] did not report the information on the gender of the patients). Among all 2049 patients, 115 received ceftriaxone, 267 were treated with doxycycline/tetracycline and 1667 with penicillin. Single dose benzathine penicillin G (BenPen) was prescribed as the comparator in most of the included studies [28, 30, 31, 33–37]. Other comparators included two doses of BenPen [32], three doses of BenPen [28, 33], clemizole penicillin G [29, 34], penicillin G [34], procaine penicillin G with aluminum stearate, and aqueous procaine penicillin G [36]. Characteristics of the included studies are summarized in Table 1.

**Table 1:** Summary of the characteristics of the 10 trials included in this research.

First author	Year	Study type	Stage of syphilis	Nontreponema antigen test	Intervention/No. of cases	Comparator/No. of cases	Regimen
Tsai	2014	Retrospective cohort	Primary, secondary and early latent syphilis	RPR	Doxyc/91	1 x BenPen/271	Doxyc (a dose of 100 mg twice daily for 14 days orally)
							BenPen (a single dose of 2.4 MU i.m.)
Li	2014	Retrospective cohort	Primary, secondary and early latent syphilis	RPR	Doxyc or tetra/35	2 x BenPen/606	Doxyc (100 mg orally twice a day for 14 days) or tetracycline (500 mg orally, 4 times a day for 14 days)
							BenPen (two doses of 2.4 MU i.m.)
Psomas	2012	Retrospective cohort	Primary, secondary and early latent syphilis	VDRL	Ceftr/49	1 x BenPen/10	Ceftr (1 or 2 g i.m. daily dose for 14 to 21 days)
						2 x BenPen/18	BenPen (1, 2, or 3 i.m. in a single daily dose of 2.4 MU)
					Doxyc/15	3 x BenPen/17	Doxyc (100 mg orally, 2 or 3 times daily, for 14 to 21 days)
						NR/7	
Spornraft-Ragaller	2011	Retrospective cohort	Primary, secondary and early latent syphilis	VDRL	Ceftr/12	1 x BenPen/8	Ceftr (i.v. 2g for 10–14 days or 2g for 21 days or 1g for 14 days)
						Clemizole penicillin G/2	BenPen (2, or 3 i.m. in a single daily dose of 2.4 MU) or clemizole penicillin G (1 MU i.m. daily for 14 or 21 days) or penicillin G (i.v. 3 x 10 MU daily for 21 days)
						Penicillin G/2	
Potthoff	2009	RCT	Primary, secondary and early latent syphilis	NR	Ceftr/27	1 x BenPen/30	Ceftr (1g i.v. for 10 days)
						3 x BenPen/35	BenPen (2.4 MU. i.m. or 3 x 2,4 MU i.m.)
Wong	2008	Retrospective cohort	Primary syphilis	RPR	Doxyc or tetra/25	1 x BenPen/420	Doxyc (100 mg orally, twice daily for 14days) or tetra (500 mg orally, 4 times daily for 14 days)
							BenPen (2.4 MU in a single dose i.m.)
Ghanem	2006	Retrospective cohort	Primary, secondary and early latent syphilis	RPR	Doxyc/34	1 x BenPen/73	Doxyc (100 mg orally twice daily for 14 days)
							BenPen (a single, i.m. 2.4 MU dose)
Schofer	1989	RCT	Primary and secondary syphilis	VDRL	Ceftr/14	Clemizole penicillin G/14	Ceftr (4 × 1 g i.m. every 2 days)
							Clemizole penicillin G (1 MU i.m. daily for 15 days)
Moorthy	1987	RCT	Primary syphilis	VDRL	Ceftr/13	1 x BenPen/4	Ceftr (3 g in a single i.m. or 2 g i.m. daily for two days or 2 g i.m. daily for five days)
							BenPen (2.4 MU in a single i.m.)
Schroeter	1972	Prospective cohort	Primary and secondary syphilis	VDRL	Tetra/67	1 x BenPen/55	Tetra (total 30 gm, 3 gm a day for ten days orally)
						Procaine penicillin G with aluminum stearate/54	BenPen (2.4 MU in a single dose i.m.) or procaine penicillin G with aluminum stearate (2.4 at first session, 1.2 at two subsequent sessions at three-day intervals, total 4.8 MU) or aqueous procaine penicillin G (600,000 units daily for total of 4.8 MU)
						Aqueous procaine penicillin G/41	

NR, not reported; MU, million units; i.m. intramuscular injection; i.v. intravenous injection; gm, gram; BenPen, benzathine penicillin G; Ceftr, ceftriaxone; Doxyc, doxycycline; Tetra, tetracycline; RCT, randomized controlled trial; RPR, rapid plasma regain test; VDRL, Venereal Disease Research Laboratory test

### Quality assessment

**Fig 2:**
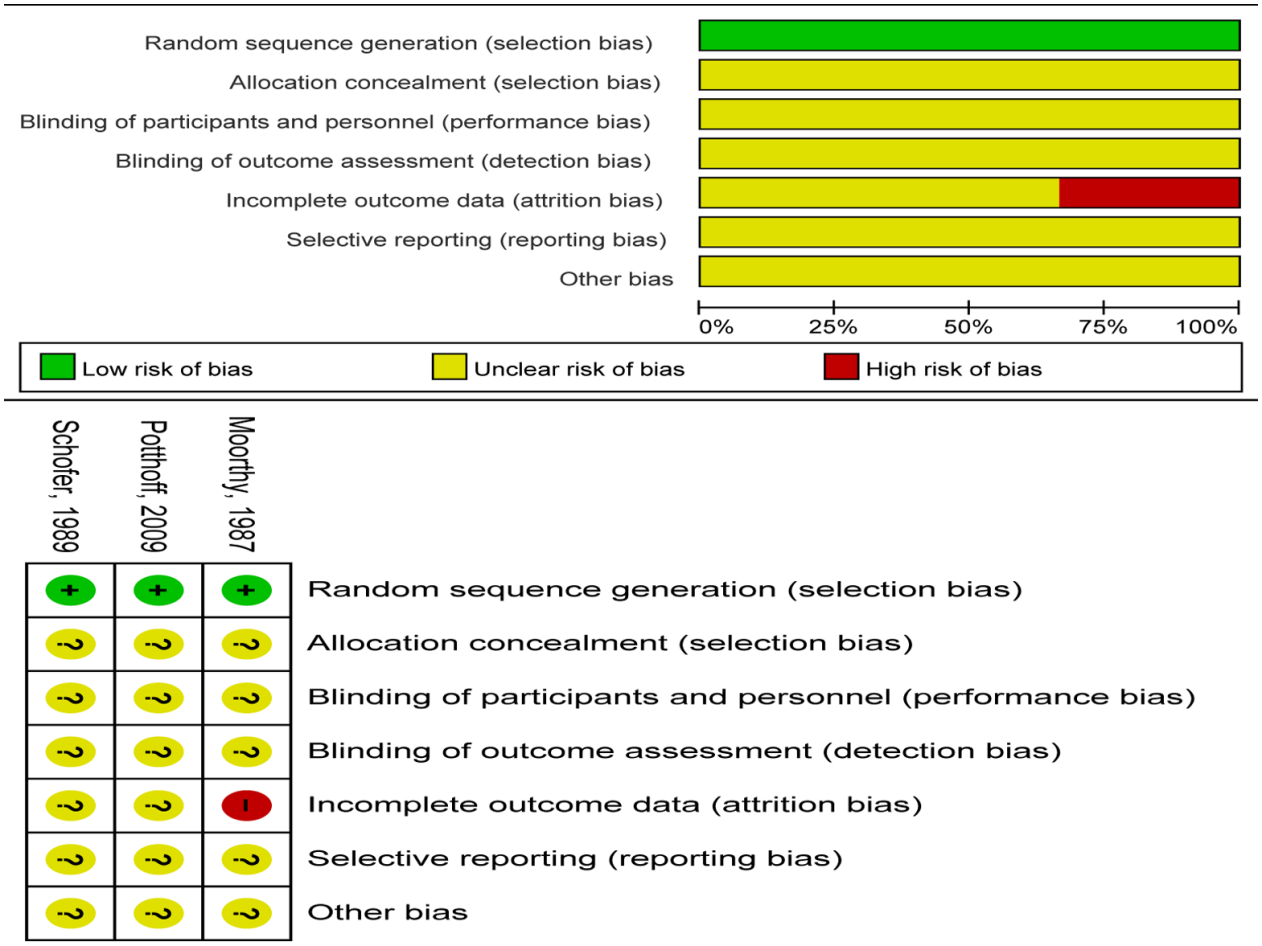
Summary diagram of risk of bias percentile chart for RCTs.

All three included RCTs mentioned randomization, but not allocation concealment and blind method; one of them had incomplete outcome data (Fig 2). On the other hand, the included observational studies met most of the quality assessment criteria, and two of them were not controlled for potential confounding factors by matching (Table 2).

**Table 2:** Results of quality assessment using Newcastle-Ottawa scale for cohort studies.

Study	Selection				Comparability	Outcome			quality score
	Representativeness of the exposed cohort	Selection of the non-exposed cohort	Ascertainment of exposure	Demonstration that outcome of interest was not present at start of study	Comparability of cohorts on the basis of the design or analysis	Assessment of outcome	Was follow-up long enough for outcomes to occur	Adequacy of follow up of cohorts	
**Tsai 2014**	**0**	**1**	**1**	**0**	**2**	**1**	**1**	**1**	**7**
**Li 2014**	**0**	**1**	**1**	**0**	**2**	**1**	**0**	**1**	**6**
**Psomas 2012**	**0**	**1**	**1**	**0**	**0**	**1**	**1**	**1**	**5**
**Spornraft-Ragaller 2011**	**0**	**1**	**1**	**0**	**2**	**1**	**1**	**1**	**7**
**Wong 2008**	**0**	**1**	**1**	**0**	**2**	**1**	**1**	**1**	**7**
**Ghanem 2006**	**0**	**1**	**1**	**0**	**2**	**1**	**1**	**1**	**7**
**Schroeter 1972**	**0**	**1**	**1**	**1**	**0**	**1**	**1**	**1**	**6**

### The median time of serological response

Of all 10 included studies, three referred to the median time of serological response, of which two compared doxycycline/tetracycline treatment with penicillin treatment [35, 37] and one involving all three treatments (ceftriaxone, doxycycline/tetracycline, and penicillin) [33]. As a result, statistically significant differences in the median time of serological response were not observed between penicillin and the alternative treatments in these studies.

### Qualitative analysis for serological response rates at 6-month follow-up

Five included studies described the results of serological tests at 6-month follow-up (Table 3), with two comparing the efficacy of ceftriaxone and penicillin [29, 30] and three comparing the efficacy of doxycycline/tetracycline and penicillin [31, 32, 36]. All of these studies except the one by Moorthy *et al*. showed that penicillin consistently achieved a higher level of efficacy than the alternative antibiotics, but their differences were not statistically significant (Table 3).

**Table 3:** Summary of data on serological response rates of interventions at 6-month follow-up.

Study	Intervention	Response	Total patients	Response rate	*P* value
**Tsai 2014**	**Doxycycline**	**78**	**123**	**63.4%**	**0.094**
	**Penicillin**	**196**	**271**	**72.3%**	
**Li 2014**	**Doxycycline/tetracycline**	**29**	**35**	**82.9%**	**0.157**
	**Penicillin**	**554**	**606**	**91.4%**	
**Schofer 1989**	**Ceftriaxone**	**5**	**6**	**83.3%**	**1.000**
	**Penicillin**	**2**	**2**	**100%**	
**Moorthy 1986**	**Ceftriaxone**	**11**	**13**	**84.6%**	**1.000**
	**Penicillin**	**4**	**5**	**80%**	
**Schroeter 1972**	**Tetracycline**	**82**	**87**	**94.3%**	**0.592**
	**Penicillin**	**191**	**198**	**96.6%**	

### A head to head meta-analysis of serological response rates at 12-month follow-up

Nine studies depicted the results of serological tests at 12-month follow-up [28–31, 33–37] (Table 4). The results of direct meta-analysis indicated that the three interventions resulted in similar serological response rates (penicillin vs. doxycycline/tetracycline RR = 0.98, 95%CI 0.78–1.23; penicillin vs. ceftriaxone RR = 1.01, 95%CI 0.90–1.14; doxycycline/tetracycline vs. ceftriaxone RR = 0.97, 95%CI 0.58–1.61) (Table 5).

**Table 4:** Summary of data on serological response rates of interventions at 12-month follow-up.

Study	Intervention	Response	Total patients	Response rate	*P* value
**Tsai 2014**	**Doxycycline**	**60**	**91**	**65.9%**	**0.681**
	**Penicillin**	**185**	**271**	**68.3%**	
**Psomas 2012**	**Doxycycline**	**11**	**15**	**73.3%**	**0.928**
	**Ceftriaxone**	**38**	**49**	**77.6%**	
	**Penicillin**	**39**	**52**	**75.0%**	
**Spornraft-Ragaller 2011**	**Ceftriaxone**	**11**	**12**	**91.7%**	**1.000**
	**Penicillin**	**11**	**11**	**100%**	
**Potthoff 2009**	**Ceftriaxone**	**16**	**27**	**59.3%**	**0.542**
	**Penicillin**	**34**	**65**	**52.3%**	
**Wong 2008**	**Doxycycline/tetracycline**	**25**	**25**	**100%**	**1.000**
	**Penicillin**	**409**	**420**	**97.4%**	
**Ghanem 2006**	**Doxycycline**	**34**	**34**	**100%**	**0.399**
	**Penicillin**	**69**	**73**	**94.5%**	
**Schofer 1989**	**Ceftriaxone**	**5**	**5**	**100%**	-
	**Penicillin**	**7**	**7**	**100%**	
**Moorthy 1986**	**Ceftriaxone**	**12**	**13**	**92.3%**	**1.000**
	**Penicillin**	**4**	**4**	**100%**	
**Schroeter 1972**	**Tetracycline**	**61**	**67**	**91.0%**	**0.357**
	**Penicillin**	**143**	**150**	**95.3%**	

**Table 5:** Results of the head to head meta-analysis on serological response at 12-month follow-up.

Comparison of interventions	No. of studies	RR(95%CI)	Heterogeneity	
			*P* value	*I*^*2*^(%)
**Penicillin vs. ceftriaxone**	**5**	**1.01(0.90–1.14)**	**0.998**	**0**
**Penicillin vs. doxycycline/tetracycline**	**5**	**0.98(0.78–1.23)**	**0.999**	**0**
**Doxycycline/tetracycline vs. ceftriaxone**	**1**	**0.97(0.58–1.61)**	-	-

RR, risk ratio; CI, confident interval

### A NMA for serological response rates at 12-month follow-up

**Fig 3:**
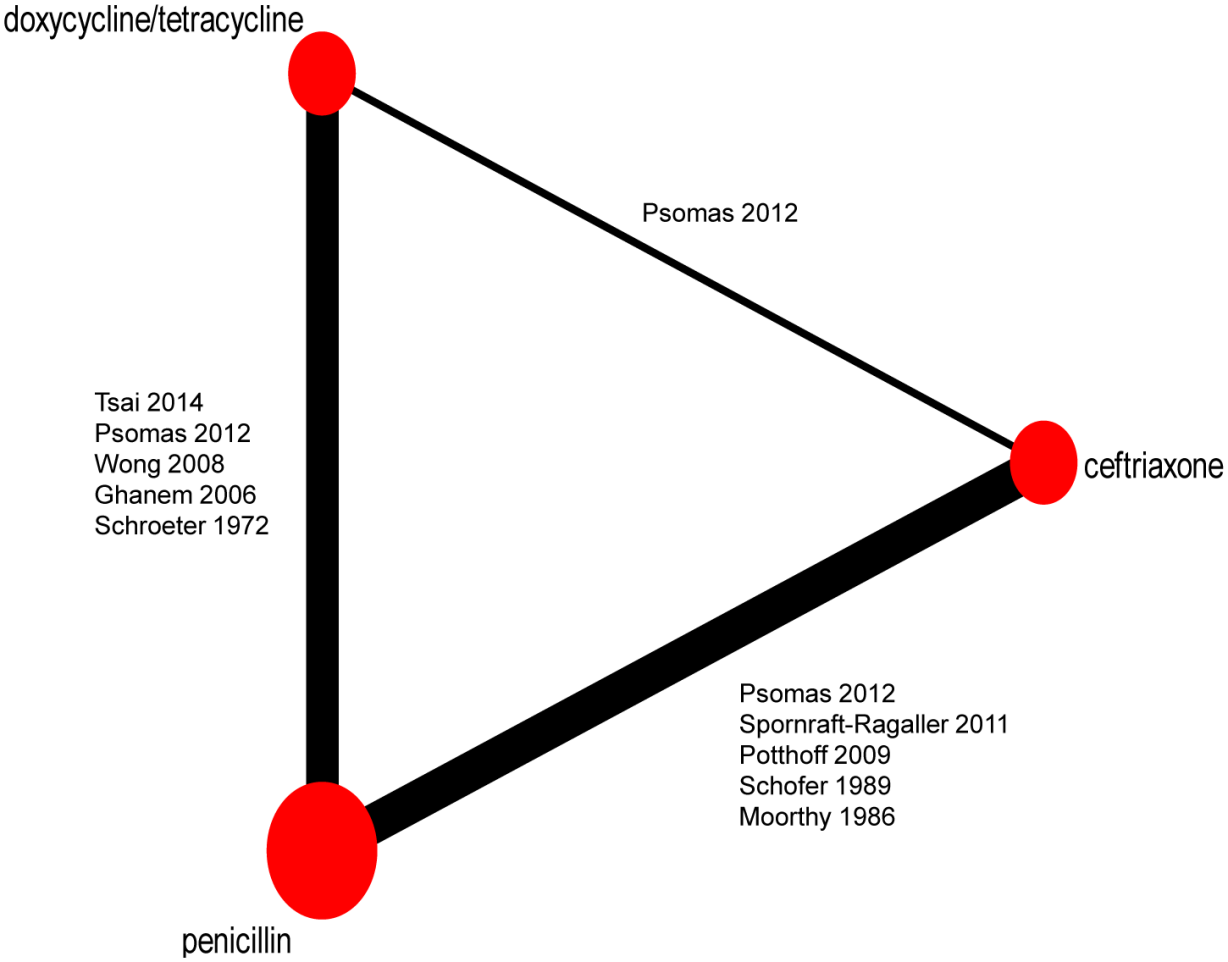
The network diagram of eligible studies.

**Fig 4:**
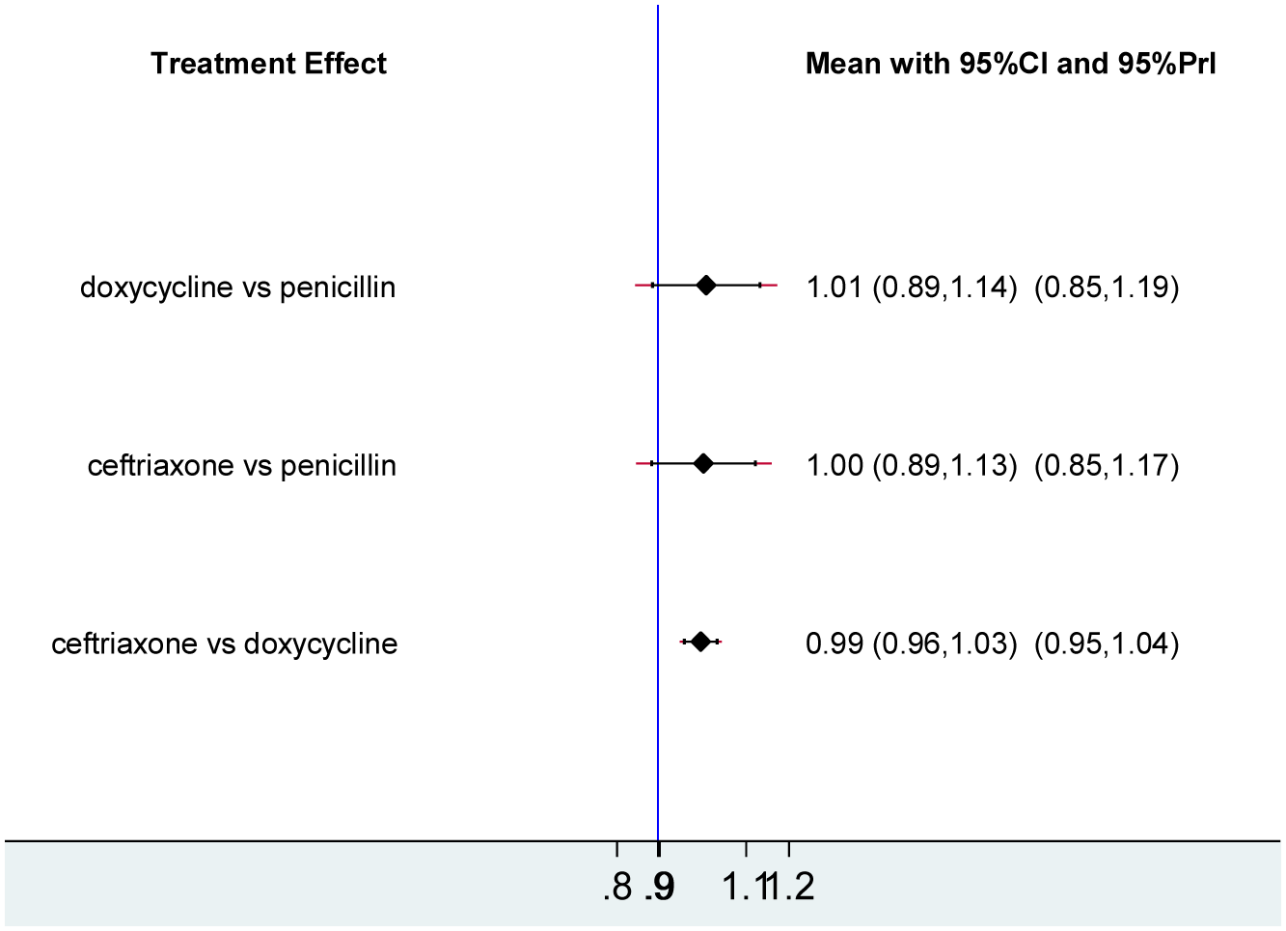
Summary of the network meta-analysis on estimates for the serological response rates at 12-month follow-up. CI, confident interval; PrI, prediction interval.

**Fig 5:**
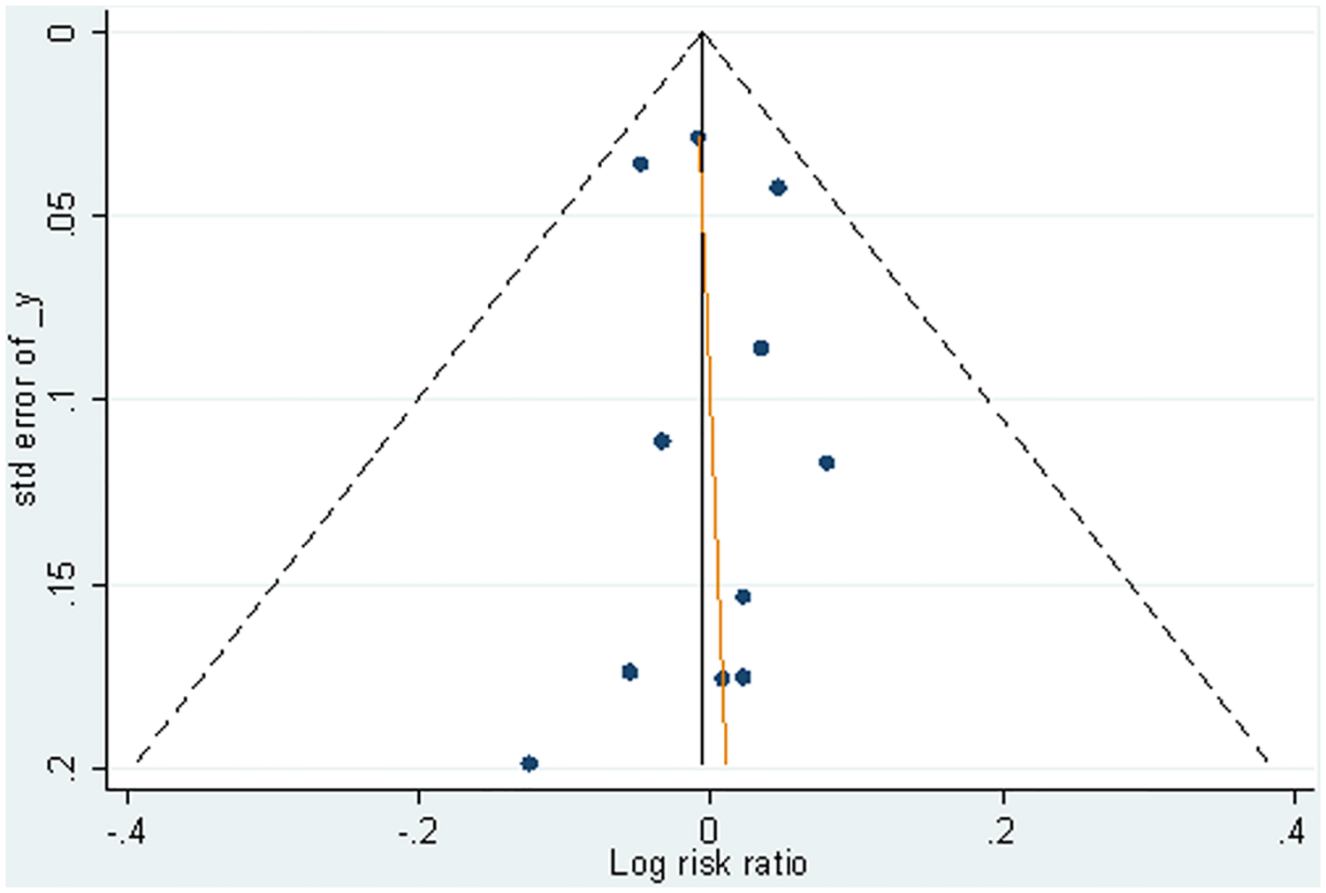
Funnel plot of the included studies reporting on the serological response at 12-month follow-up. Funnel plot provides a scatter diagram which could visually assess publication bias. In the absence of bias the plot will resemble a symmetrical inverted funnel.

Fig 3 is the network diagram of included studies. In line with the above results of the head to head meta-analysis in Table 5, the pooled RR for doxycycline/tetracycline vs. penicillin was 1.01 (95%CI 0.89–1.14, 95%PrI 0.85–1.19), for ceftriaxone vs. penicillin was 1.00 (95%CI 0.89–1.13, 95%PrI 0.85–1.17), and for ceftriaxone vs. doxycycline/tetracycline was 0.99 (95%CI 0.96–1.03, 95%PrI 0.95–1.04) (Fig 4). There was no evidence of inconsistency between the direct and the indirect comparison, which was assessed using node-splitting method. Also, no significant heterogeneity was found among individual studies based on the Q and *I*^*2*^ test (*P* = 1.000, *I*^*2*^ = 0). Fig 5 showed a symmetric funnel plot, which visually illustrated the absence of publication bias. In line with this, there was no significant publication bias for included studies (Begg Mazumdar test *P* = 0.392; Egger’s test bias = 0.11, *P* = 0761). Moreover, sensitivity analysis using exclusion of any single study did not show any substantial changes in the pooled RR. It confirmed the robustness of our findings.

**Fig 6:**
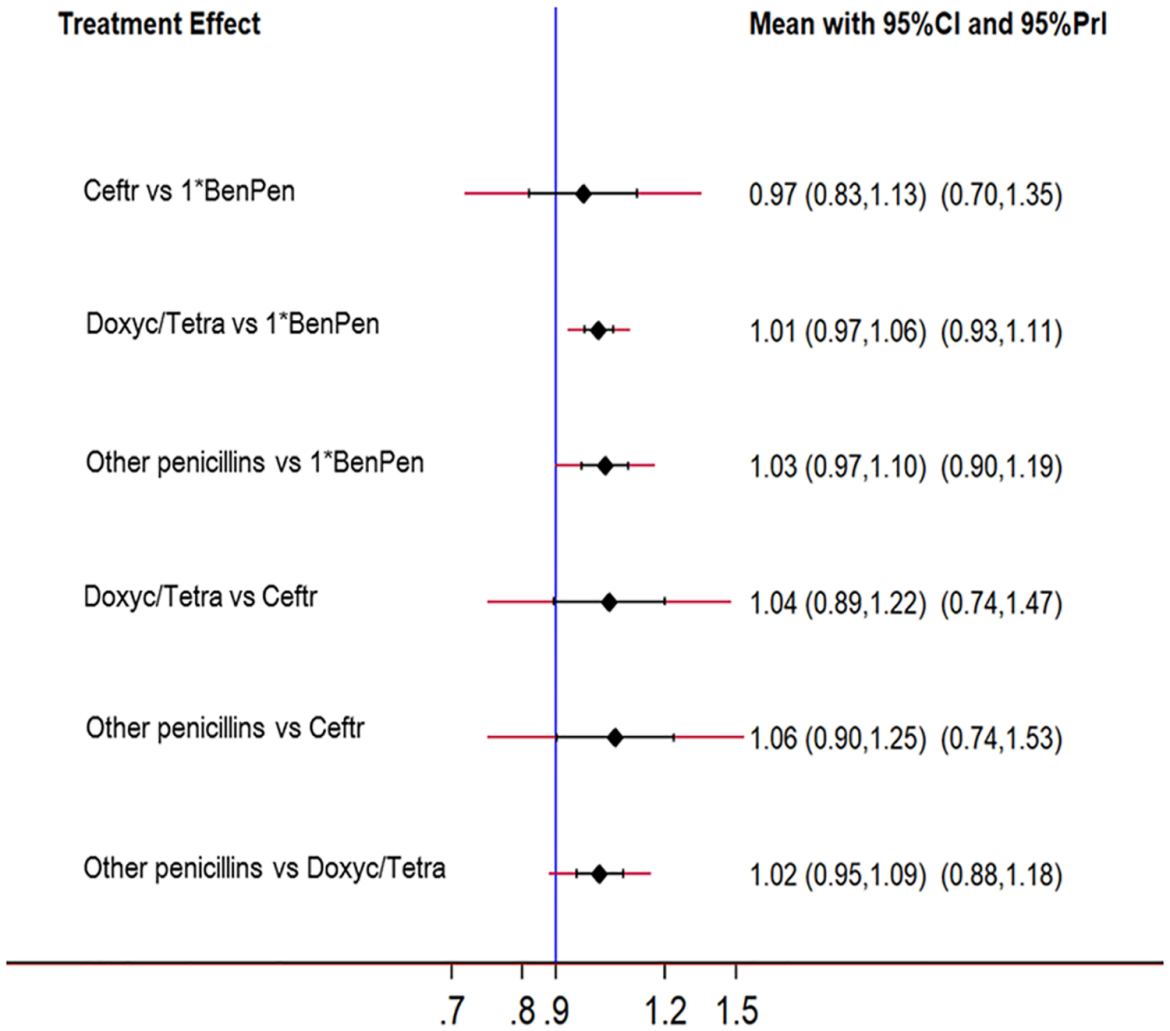
Summary of the network meta-analysis on estimates based on different penicillin regimens. BenPen, benzathine penicillin G; Ceftr, ceftriaxone; Doxyc, doxycycline; Tetra, tetracycline.

To further evaluate the efficacy of penicillin and alternative drugs, we classified penicillin into single dose BenPen and other types (three doses of BenPen, clemizole penicillin G, penicillin G, procaine penicillin G with aluminum stearate, and aqueous procaine penicillin G) according to the data of included studies [28–31, 35–37]. Based on the different penicillin regimens, a NMA was conducted, which showed that no significant differences existed in serological response rate between any two treatments (ceftriaxone vs. single dose BenPen RR = 0.97, 95%CI 0.83–1.13, 95%PrI 0.70–1.35; doxycycline/tetracycline vs. single dose BenPen RR = 1.01, 95%CI 0.97–1.06, 95%PrI 0.93–1.11; other penicillin regimens vs. single dose BenPen RR = 1.03, 95%CI 0.97–1.10, 95%PrI 0.90–1.19; doxycycline/tetracycline vs. ceftriaxone RR = 1.04, 95%CI 0.89–1.22, 95%PrI 0.74–1.47; other penicillin regimens vs. ceftriaxone RR = 1.06, 95%CI 0.90–1.25, 95%PrI 0.74–1.53; other penicillin regimens vs. doxycycline/tetracycline RR = 1.02, 95%CI 0.95–1.09, 95%PrI 0.88–1.18) (Fig 6). This suggested that the similarities in serological response existed not only between penicillin and alternative antibiotics but also between single dose BenPen and other penicillin regimens, which are the evidence supporting the present guidelines recommending treatment of early syphilis with single dose BenPen [6–8].

### A head to head meta-analysis of treatment failure rates at 12-month follow-up

Seven included studies reported treatment failure at 12-month follow-up [29–31, 34–37]. Since only 30 patients were treated with ceftriaxone in these studies, we analyzed the treatment failure rates with a head to head meta-analysis. As a result, the serological failure rate at 12-month follow-up was significantly lower in penicillin recipients than in doxycycline/tetracycline recipients (RR = 0.58, 95%CI 0.38–0.89), while significant differences in this rate were not observed between treatments penicillin and ceftriaxone (RR = 0.92, 95%CI 0.12–6.93) (Table 6).

**Table 6:** Results of the head to head meta-analysis on treatment failure at 12-month follow-up.

Comparison of interventions	No. of studies	RR (95%CI)	Heterogeneity	
			*P* value	*I*^*2*^(%)
**Penicillin vs. doxycycline/tetracycline**	**4**	**0.58 (0.38–0.89)**	**0.468**	**0**
**Penicillin vs. ceftriaxone**	**3**	**0.92 (0.12–6.93)**	**0.992**	**0**

RR, risk ratio; CI, confident interval

## Discussion

To our knowledge, the present study is the first NMA simultaneously comparing multiple interventions by integration of the direct and indirect evidence in syphilis therapy. Traditionally, NMAs only incorporate data from RCTs because confounding factors could be balanced by randomized means, but recently, an increasing number of NMAs have combined RCTs with observational studies [38–41]. Because RCTs for assessing the efficacy of penicillin alternatives in treating syphilis are scant, we combined RCTs and observational studies in our study; such combination was proved to be valid because of no evidence of heterogeneity in included studies, the consistent results of direct and network meta-analysis and the robustness of pooled estimates.

Our results showed that all three treatments (penicillin, ceftriaxone, and doxycycline/tetracycline) did not differ significantly in serological response rates at 6-month and 12-month follow-up and in median time of serological response. Furthermore, the direct meta-analysis and the NMA consistently demonstrated that penicillin and the alternative treatments resulted in similar serological response rates at 12-month follow-up. On the other hand, treatment with doxycycline/tetracycline led to a significantly higher serological failure rate at 12-month follow-up than treatment with penicillin, with a ratio of 1/0.58, whereas treatments ceftriaxone and penicillin had similar failure rates, which was in accord with a previous meta-analysis [16]. Despite the similarities between penicillin and the alternatives in serological response rate, their significant differences in treatment failure rate deserve our attention as it could bring severe consequences. The differences in treatment failure rate among these drugs may stem from their dissimilarities in targets, the mechanisms of action, and the compliance degree of patients. Doxycycline is a tetracycline derivative with better oral bioavailability and fewer gastrointestinal side effects [42]; its low cost and oral administration have logistical advantages, which, however, may reduce the compliance degree of patients due to lack of supervision. On the other hand, like penicillin, ceftriaxone requires parenteral administration, which may improve the compliance degree of patients, but it is costly and its administration may bring the risk of the cross-sensitivity with penicillin; hence, skin test should be done before its administration. Overall, this study signifies that ceftriaxone is as effective as penicillin in treating early syphilis, while doxycycline/tetracycline is less effective than penicillin with regard to treatment failure rate.

Our study has the following strengths. The efficacy of penicillin and alternative drugs for early syphilis was assessed by serological response and treatment failure rate. In methodology, NMA was performed for the first time to simultaneously compare the efficacy of ceftriaxone, doxycycline/tetracycline, and penicillin in addition to direct meta-analysis. Until now, there has been no documented meta-analysis on the efficacy of doxycycline/tetracycline and comparison of the efficacy of multiple drugs in treating early syphilis, particularly for penicillin alternative drugs. Our findings not only fill in these gaps, but also provide a useful guidance for choosing the optimal alternative drug clinically.

The present study has some limitations. Firstly, because RCTs and observational studies were combined in the analysis, the results should be interpreted with caution, although evidence of heterogeneity was not found. Secondly, due to a relatively low number of patients receiving ceftriaxone or doxycycline/tetracycline in the included studies, we could not determine whether the differences in the efficacy of different treatments were attributed to varied treatment regimens, or to different syphilis stages such as primary, secondary or early latent. Thirdly, the regimens of interventions varied in different trials, which made it impossible for us to determine the optimal dose and treatment course for each tested drug. Finally, we were unable to clearly distinguish between treatment failure and reinfection, which might affect the assessment of treatment failure.

Based on the obtained results, we conclude that the efficacy of ceftriaxone is equivalent to that of penicillin in treating early syphilis in terms of serological response rate and treatment failure rate. Compared to doxycycline/tetracycline, ceftriaxone is more suitable for use as a substitute for penicillin in the treatment of early syphilis. If patients are given doxycycline/tetracycline, a careful follow-up should be conducted, so that treatment failure could be identified early. Besides that, it is necessary to develop high-quality, large-scale RCTs to verify the efficacy of ceftriaxone and doxycycline/tetracycline in treating early syphilis.

## Supporting information

S1 Table PRISMA 2009 checklist of the paper. (DOC)

S2 Table PubMed search strategy and result. (DOCX)
