# Supplementary material for: Comparison of efficacy of treatments for early syphilis: A systematic review and network meta-analysis of randomized controlled trials and observational studies
Source: PLoS One. 2017 Jun 28;12(6):e0180001. doi: 10.1371/journal.pone.0180001 (PMC5489196; doi:10.1371/journal.pone.0180001)
Supplement: S2 Table — (DOCX) [file pone.0180001.s002.docx]

S2 Table. PubMed search strategy and result

| **Search** | **Query** | **Items found** | **Time(June 30, 2016)** |
| --- | --- | --- | --- |
| #1 | Search (("Syphilis"[Mesh]) OR ((Great Pox[Title/Abstract]) OR Pox, Great[Title/Abstract])) | 25719 | 20:46:58 |
| #2 | Search ("Tetracycline"[Mesh]) OR ((((((((((Tetrabid[Title/Abstract]) OR 4-Epitetracycline[Title/Abstract]) OR 4 Epitetracycline[Title/Abstract]) OR Topicycline[Title/Abstract]) OR Achromycin V[Title/Abstract]) OR Hostacyclin[Title/Abstract]) OR Tetracycline Hydrochloride[Title/Abstract]) OR Tetracycline Monohydrochloride[Title/Abstract]) OR Sustamycin[Title/Abstract]) OR Achromycin[Title/Abstract]) | 18957 | 20:51:41 |
| #3 | Search (("Ceftriaxone"[Mesh]) OR ((((((((((((((((((((((((((((((((((((((((Ceftriaxon[Title/Abstract]) OR Cefatriaxone[Title/Abstract]) OR Terbac[Title/Abstract]) OR Syntex Brand of Ceftriaxone Sodium[Title/Abstract]) OR Ceftrex[Title/Abstract]) OR Columbia Brand of Ceftriaxone[Title/Abstract]) OR Ceftriaxon Curamed[Title/Abstract]) OR Curamed Brand of Ceftriaxone Sodium[Title/Abstract]) OR Ceftriaxon Hexal[Title/Abstract]) OR Hexal Brand of Ceftriaxone Sodium[Title/Abstract]) OR Ceftriaxona Andreu[Title/Abstract]) OR Boehringer Mannheim Brand of Ceftriaxone Sodium[Title/Abstract]) OR Ceftriaxona LDP Torlan[Title/Abstract]) OR Inibsa Brand of Ceftriaxone Sodium[Title/Abstract]) OR Ceftriaxone Irex[Title/Abstract]) OR Irex Brand of Ceftriaxone[Title/Abstract]) OR Ceftriaxone Sodium[Title/Abstract]) OR Sodium, Ceftriaxone[Title/Abstract]) OR Ceftriaxone, Disodium Salt, Hemiheptahydrate[Title/Abstract]) OR Ceftriaxone, Disodium Salt[Title/Abstract]) OR Ceftriaxone Sodium, Anhydrous[Title/Abstract]) OR Anhydrous Ceftriaxone Sodium[Title/Abstract]) OR Lendacin[Title/Abstract]) OR Longacef[Title/Abstract]) OR Longaceph[Title/Abstract]) OR Ro13-9904[Title/Abstract]) OR Ro13 9904[Title/Abstract]) OR Ro139904[Title/Abstract]) OR Ro-13-9904[Title/Abstract]) OR Ro 13-9904[Title/Abstract]) OR Ro 13 9904[Title/Abstract]) OR Ro 139904[Title/Abstract]) OR Rocephin[Title/Abstract]) OR Roche Brand of Ceftriaxone Sodium[Title/Abstract]) OR Rocefin[Title/Abstract]) OR Rocephine[Title/Abstract]) OR Hoffman-La Roche Brand of Ceftriaxone Sodium[Title/Abstract]) OR Hoffman La Roche Brand of Ceftriaxone Sodium[Title/Abstract]) OR Rocefalin[Title/Abstract]) OR Tacex[Title/Abstract])))) | 5233 | 20:56:17 |
| #4 | Search (("Doxycycline"[Mesh]) OR (((((((((((((((((((((((((((((((((((((Alpha-6-Deoxyoxytetracycline[Title/Abstract]) OR Alpha 6 Deoxyoxytetracycline[Title/Abstract]) OR Vibravenos[Title/Abstract]) OR BU-3839T[Title/Abstract]) OR BU 3839T[Title/Abstract]) OR BU3839T[Title/Abstract]) OR Doryx[Title/Abstract]) OR Doxycycline Calcium[Title/Abstract]) OR Doxycycline Calcium Salt AND (1 : 2) AND Title/Abstract) OR Doxycycline Hyclate[Title/Abstract]) OR Hyclate, Doxycycline[Title/Abstract]) OR Doxycycline Hemiethanolate[Title/Abstract]) OR Hemiethanolate, Doxycycline[Title/Abstract]) OR Doxycycline Monohydrate[Title/Abstract]) OR Doxycycline Monohydrochloride, 6-epimer[Title/Abstract]) OR Doxycycline Monohydrochloride, 6 epimer[Title/Abstract]) OR Doxycycline Monohydrochloride, Dihydrate[Title/Abstract]) OR Dihydrate Doxycycline Monohydrochloride[Title/Abstract]) OR Monohydrochloride, Dihydrate Doxycycline[Title/Abstract]) OR Doxycycline Phosphate AND (1 : 1) AND Title/Abstract) OR Doxycycline-Chinoin[Title/Abstract]) OR Doxycycline Chinoin[Title/Abstract]) OR Hydramycin[Title/Abstract]) OR Oracea[Title/Abstract]) OR CollaGenex Brand of Doxycycline[Title/Abstract]) OR Doxycycline CollaGenex Brand[Title/Abstract]) OR Periostat[Title/Abstract]) OR Vibra-Tabs[Title/Abstract]) OR Vibra Tabs[Title/Abstract]) OR VibraTabs[Title/Abstract]) OR Vibramycin[Title/Abstract]) OR Vibramycin Novum[Title/Abstract]) OR Novum, Vibramycin[Title/Abstract]) OR Atridox[Title/Abstract]) OR BMY-28689[Title/Abstract]) OR BMY 28689[Title/Abstract]) OR BMY28689[Title/Abstract])))) | 8240 | 21:03:20 |
| #5 | Search ((clinical[tiab] AND trial[tiab]) OR "clinical trials as topic"[mesh] OR "clinical trial"[pt] OR random*[tiab] OR "random allocation"[mesh] OR "therapeutic use"[sh]) | 4596008 | 21:08:16 |
| #6 | Search ((((#2) OR #3) OR #4) AND #1) AND #5 | 275 | 21:14:13 |
